# Resolving the vertical versus horizontal conundrum: the potential of a “next generation” community health worker program to accelerate progress in TB control and at the same time strengthen primary health care

**DOI:** 10.3389/fpubh.2026.1815283

**Published:** 2026-04-29

**Authors:** Henry B. Perry, Akramul Islam, Shayla Islam, Farhana Nishat Seheli, Saifur Reja, Mirlene I. Perry, Krupa K. Patel, Asif Saleh, A. Mushtaque R. Chowdhury

**Affiliations:** 1 Department of International Health, Johns Hopkins Bloomberg School of Public Health, Baltimore, MD, United States; 2 BRAC, Dhaka, Bangladesh; 3 Duke University School of Nursing, Durham, NC, United States; 4 Duke Global Health Institute, Durham, NC, United States; 5 CBIO Initiatives for Global Health, Durham, ND, United States; 6 BRAC University, Dhaka, Bangladesh

**Keywords:** BRAC, community health worker, community-based primary health care, tuberculosis, primary health care, vertical versus horizontal programming in global health

## Abstract

Building on the experience of the community health worker (CHW) program at BRAC, the largest NGO in Bangladesh and also in the world, as well as on the experience of other successful CHW programs, a “next generation” of CHW programs is being envisioned in which a CHW would visit every home every month. Among many other activities they would carry out, CHWs would screen for symptoms of TB, obtain sputum specimens that would be collected by a supervisor, have them read at a local government laboratory, and then ensure treatment supervision for those who test positive. This is but one example of the power of a full-throated integrated CHW program that could be implemented in low- and lower middle-income countries at a cost of $26 per capita per year and generate over 20 years a 13:1 return on investment. Unfortunately, at present progress in achieving the health-related Sustainable Development Goals is far below what is needed in order to achieve in 2030 the success that was envisioned. Investing in stronger CHW programs and strengthening community-based primary health care has the potential to accelerate progress and at the same time resolve the vertical versus horizontal conundrum.

## Introduction

In this paper, we highlight the potential of a “next generation” community health worker (CHW) program to strengthen tuberculosis (TB) control within the context of an integrated, community-based primary health care (CBPHC) program. The vision for this “next generation” CHW program comes in part from BRAC’s CHW program, which has embedded within it an approach to TB control that has had national impact in Bangladesh. This approach holds great potential, particularly in the current era of reduced donor support and stagnation in achievement of the Sustainable Development Goals (SDGs), for controlling TB and other priority conditions throughout the world while at the same time reducing health care expenses. BRAC is the largest NGO in Bangladesh, reaching 110 million people with its services, and the largest NGO in the world, engaging 145 million people in 11 different countries (1).

A “next generation” CHW program has been proposed for low- and lower-middle income countries that has as its foundation a CHW visit to every home every month – to provide a range of services, including screening for symptoms of TB, sending sputum specimens from symptomatic patients for laboratory examination, and providing supervised treatment with Directly Observed Treatment Short Course (DOTS) (2). Such a “next generation” CHW program has the potential to not only accelerate progress in TB control but also in all the health-related SDGs (2). 2026 is beyond the halfway point toward achieving the SDGs established in 2015 to be met by 2030. The world has fallen far behind in its progress. Only 17% of SDG targets are on track, while nearly half are showing minimal or moderate progress and over one third have stalled or even regressed (3). Declines in maternal mortality have stalled: between 2015 and 2020, the global maternal mortality ratio declined minimally from 227 to 223 maternal deaths per 100,000 live births (3). Progress in reducing under-5 mortality needs to accelerate in 59 countries, nearly 75% of which are located in sub-Saharan Africa (3). Immunization coverage rates have not fully recovered from pandemic-era declines. The world is off track in reaching its goal for reducing the number of new HIV infections, for reducing the incidence and number of deaths from TB, and for ending malaria by 2030 (3).

## TB as a global health priority

About a quarter of the world’s population is estimated to have latent/asymptomatic TB infection (4). In 2024, an estimated 10.7 million people fell ill with symptomatic TB (4), and 1.2 million people died from TB, including 150,000 who also had HIV (4). Those with latent TB who develop diminished immunocompetence, such as those with HIV infection, are predisposed to activating their latent infection and developing an active case. Despite improvements in detection, 8.3 million people were reported as newly diagnosed with TB in 2024 (about 78% of the estimated number of incident cases), leaving approximately 2.4 million cases not diagnosed with TB or not officially reported to national authorities in 2024 (4). Poor housing, crowded living spaces, limited access to screening and treatment, suboptimal medication adherence, along with stigma associated with the disease are all barriers to the detection, treatment, and cure of TB. Among people living with HIV in low-income countries, TB is a leading cause of death (5). Other comorbidities such as diabetes and malnutrition exacerbate TB and negatively affect treatment outcomes. Incomplete treatment or loss to follow-up of treatment is a major contributor to the development of strains of drug resistant TB.

## The contributions that CHWs can make to TB control

TB control rests on early detection of cases, treatment with an appropriate combination of oral medications (with priority given to completion of treatment since incomplete treatment or loss to follow-up increases the risk of development of drug-resistant strains of TB), systematic contact investigation, and provision of preventive treatment to eligible contacts (as well as to people living with HIV). Traditionally, TB control programs have relied on a passive approach to case detection, using public media messages to raise awareness about symptoms of TB and the importance of coming to a government clinic for sputum testing. Then, for those who test positive, patients are given medication for a limited period of time and told to come back to the clinic for follow up and replenishment of their medications. In these traditional TB control programs, special TB workers, who are field staff assigned for local TB control within the vertical TB control program, are dispatched to track down contacts and link them to prophylactic treatment if they are asymptomatic or begin them on full treatment if they have active infection. Even in most countries with CHW programs, contact tracing is still carried out by staff employed in the TB program rather than by CHWs. Often, contact tracing is one of the weakest parts of vertical TB control programs in resource-constrained settings, partly because of their lack of integration with primary health care (PHC) (6).

This approach is especially problematic in areas of the world where government clinics are not readily available and where people are hesitant to obtain health care at distant facilities. This is, in fact, the case in most areas of the world. As a result, this approach leads to delayed case detection, limited follow-up of contacts, high rates of treatment abandonment, further spread of the disease, and the development of cases with drug-resistant TB. Drug-resistant TB is a major challenge to cure since it involves treatment with more expensive medications for a longer period of time than is the case for those who do not have drug resistant TB.

Scale-up of diagnostics and broader access to treatment have led to progress in TB control in recent years. However, progress has been too slow globally if the 2030 SDG targets are to be reached. After decades and tens of billions of dollars invested, we have made insufficient progress in detecting cases, in improving treatment coverage, and in holding systems accountable for following up and supporting patients through the completion of their treatment (3, 4). One conclusion is now obvious: stronger community-based approaches are needed to reach a higher percentage of cases, especially cases in marginalized and vulnerable communities where geographic, economic, and social barriers prevent ready access to services.

CBPHC strengthens TB control in three important ways. First, CHWs who visit homes on a regular basis can reduce the time to diagnosis and also reduce further transmission by linking individuals promptly to TB treatment (7). Second, by performing active follow-up and providing support to people undergoing treatment for TB, CHWs can improve treatment adherence and help ensure they complete treatment. Third, at the time of home visits, CHWs can share information with all their clients about TB, its transmission, and its curability, thereby slowing transmission and reducing the number of new cases (7).

Despite these advantages, CHWs also face certain operational challenges. Some marginalized populations, including climate migrants, residents of urban slums, and people living in isolated rural areas, may remain difficult to reach through routine household visits alone. In such settings, additional outreach activities are often conducted by field staff with support from donor-funded initiatives. In addition, maintaining the quality of CHW-delivered services requires adequate supervision, periodic refresher training, and supportive program management to ensure effective service delivery.

The urgency to make the case for stronger community engagement in TB control is now made even more so by the current sharp decline in donor funding. The World Health Organization (WHO) and other global health partners have issued warnings that dwindling global financing will reverse hard-won TB control efforts (8). In its place, CHW-led integrated strategies that tackle not only TB but also HIV, maternal and child health, immunization, and rising chronic diseases will be needed. This will, we argue, make it possible to “do more with less” because of the gains from relying on lower-level, less expensive health workers to provide an increased share of the workload needed for effective health system functioning.

CHWs can help fill the gap produced by decreased funding for vertical disease programs. In addition, when effectively implemented, CHWs can provide promotive, preventive, and curative services at substantially lower costs than by expanding facility-based care (9–11). CHWs can reach populations that are vulnerable because CHWs live within the communities they serve and are trusted by them. CHWs can reach everyone through visits to all household and deliver timely care to patients who might otherwise skip or delay treatment because of the distance to a facility, transportation costs, long wait times at overcrowded facilities, and fear of disrespectful treatment.

## The proposed “next generation” of CHW programs

Universal Health Coverage and Health for All will not be achievable in low-income and lower-middle-income countries without a significant scale-up of CBPHC (12). Too many health systems continue to rely on facility-based care and hospital-centric models that fail to reach large swaths of the population due to geographic, financial, and health workforce barriers. As a result, readily preventable illness and death persist, as do high levels of health inequity. CHW programs need to be strengthened to become a pillar of the national health system rather than a stopgap measure (13).

Accordingly, a strengthened Community Platform for PHC is needed in which well-trained, salaried, supplied, and supervised CHWs perform routine household visits and maintain regular contact with families (2). This would allow CHWs to provide integrated care that extends beyond maternal and child health to include family planning; HIV; malaria; tuberculosis; screening and follow-up care for hypertension, diabetes, and other noncommunicable diseases; outbreak detection and surveillance; and registration of vital events. This holistic platform would improve continuity of care and strengthen referral systems while reaching underserved people who otherwise may never set foot in a health facility.

Working with UNICEF and an Expert Panel, a “next generation” CHW program has been envisioned and costed that would have the potential to strengthen TB control as well as address other priority health conditions (2). It would have a “dual cadre” of CHWs: one cadre of professionalized, full-time (FT) CHWs (1 for every 1,000 people) and a cadre of paid part-time (PT) CHWs working one-third time (1 for every 25 households, or every 125 people). The PT-CHWs would visit every household every month (or more frequently if needed). Each FT-CHW would be a team leader for 8 PT-CHWs, and the team would meet together monthly. The annual cost of such a generic CHW program for all low-income and lower-middle income countries is estimated to be $26 per capita, about four times the cost of the average CHW program at present (2).

This dual-cadre system is designed to balance intensive community outreach activities with effective monitoring and technical support. PT-CHWs would maintain monthly household contact and deliver basic preventive and promotive services, while FT-CHWs would provide higher-level services, e.g., prenatal care, supervision, coordination with the health center, and maintenance of referral and counter-referral linkages. Together, this structure would strengthen the connection between communities and the formal health system, enabling earlier detection of health problems, improved continuity of care, and more equitable access to essential health services. Such a dual-cadre system would make it possible to link a smaller number of more highly trained CHWs with a larger population via lower-level CHWs, thereby expanding quality access to higher-level care at a lower cost than relying on a larger number of more highly trained CHWs.

Such an investment could save millions of lives and yield a return on investment over 20 years of approximately 13:1 through reduced health-care expenditures and healthier, more productive populations (2). Beyond these benefits, such a “next generation” of CHWs could contribute to improved health literacy, trust and confidence in health systems, women’s empowerment, advancement of equity and social justice, reduced congestion at clinics and hospitals, and improved preparedness for epidemics, pandemics, and climate-related health threats (2).

## The Bangladesh government-NGO TB control program

For the past 50 years, Bangladesh has been an “exceptional health performer” in its fight against priority diseases, including TB (14, 15). In 1987, the incidence rate of TB was 870 per 100,000 people, four times the current level of 221 (16). In 1987, only 20% of cases were detected compared to 69% in 2024. The treatment success rate in 1987 was only 20% compared to 96% at present, one of the highest in the world (4, 17, 18). During the past decade (2015—2023) the number of deaths from TB has declined by 35% (18).

In 1984, BRAC initiated its first TB program in one district, Manikganj thana (district) (19). BRAC trained and supervised CHWs (*Shasthya Shebikas, or SSs*) to identify suspected cases (those with a cough for more than 2 weeks) at the time of routine monthly home visits. They collected sputum specimens that were examined at the government’s district laboratory. Then, the SSs were authorized to provide DOTS to those who were sputum positive and were provided with the necessary medications.

These SSs are engaged in multiple other activities beyond TB, primarily maternal and child work and family planning. SSs provide community education and selected PHC services through routine home visits, at which time they inquire about symptoms of TB. This enables early detection and early initiation of treatment while at the same time promoting broader awareness of TB. Emphasis is placed on treatment completion in order to achieve full recovery, prevent drug resistance, and minimize transmission (20).

These CHW activities have historically been supported through BRAC’s program resources (which are substantial as a result of its profit-making commercial enterprises) and donor-funded initiatives implemented in collaboration with the national TB program. This financing structure has enabled the scale-up of community-based TB activities while highlighting the importance of sustainable domestic financing for long-term program stability. Within Bangladesh’s health system, CHWs funded by both the government and NGOs function as part of the broader CBPHC platform, and they work in close coordination with government facilities for referral, diagnosis, and continuity of care (20, 21). The Global Fund to Fight AIDS, Tuberculosis and Malaria has been an important source of funding to the government and to NGOs (including BRAC) for TB activities.

CHWs facilitate seamless interaction between communities and the health system. Through household outreach and routine community engagement, CHWs maintain regular relationships with both community members and health facilities. These activities help enable timely case detection, treatment initiation, and follow-up care. By integrating CHWs into PHC, a stronger connection is created between health care delivery at the community and facility levels. Increased coordination across both settings helps improve treatment adherence and allows for better monitoring of patients throughout TB treatment.

With collaboration from the government, BRAC was able to expand this program to nine other districts initially, then to 120 districts by 1997 (19). At that time the prevalence of TB in BRAC-serviced sub-districts was only half of that compared to areas without the BRAC TB program (19). Other NGOs joined BRAC with similar programs for TB control. Over time, the program scaled nationwide, ultimately covering the entire country. At present, BRAC’s TB program reaches 80% of the national population of 178 million people. In 2024, it identified 313,926 cases of TB, investigated 339,836 contacts, and enrolled 136,726 in preventive treatment (22).

Based on this progress, support from the Global Fund to Fight AIDS, TB, and Malaria made it possible to establish in 2002 the Bangladesh Country Coordinating Mechanism (BCCM), comprised of 33 organizations in the public and private sectors (23). The BCCM has played a pivotal role in the implementation, monitoring, and evaluation of the country’s programs and served as a watchdog on behalf of civil society. The national TB program and NGOs, including BRAC, have followed the same government treatment guidelines and approaches. BRAC’s TB program has been able to achieve high rates of detection and cure rates at a substantially lower cost (24).

BRAC has also provided leadership in Bangladesh on addressing social and structural barriers to TB control, such as stigma and gender discrimination, through community engagement and promotion of the rights of people affected with TB (23). BRAC has provided comprehensive TB services to the hardest to reach patients in urban slums and isolated rural areas (23). Although Bangladesh has a low prevalence of HIV (0.1%), the number of detected cases of HIV is growing, posing additional challenges for TB control.

BRAC, founded in 1972 in Bangladesh, is now the world’s largest Global South-led development organization, tackling poverty through programs in health, education, microfinance, and more (1). It has a long history of gradually integrating government vertical programs with its community-level health programs led by its own CHWs—most notably for family planning and immunizations—and linking them with government vertical programs to create a win-win situation (25). Increasingly, the government’s CHWs are collaborating at the local level among themselves and with NGOs’ CHWs such as BRAC’s to ensure linkage of individuals and families with vertical health programs when appropriate (26). This rich mix of vertical and horizontal programming at the community level is one one important reason that Bangladesh has been a global leader in improving the health of its population (15, 20, 21).

## The potential of CHWs to accelerate progress in TB control globally while at the same time contributing to accelerated progress for other health-related SDGs

Meeting the 2030 global targets for TB and the other health-related SDGs will not be possible without a major paradigm shift to integrated, community-based models of PHC. Going forward, and in the face of dwindling donor budgets, countries will need to commit more domestic resources to health [as Bangladesh is now doing (27)], as well as spend these resources efficiently. Task-shifting to CHWs is one strategy to effectively extend services to underserved populations at relatively low cost. External donors can support this transition by assisting governments to identify locally appropriate and sustainable options to finance CHW platforms going forward. By moving beyond traditional disease-specific program support and prioritizing scalable, integrated community health platforms, donors and governments can strengthen TB programs while also accelerating progress toward broader SDG health targets. The evidence is increasingly clear: if designed and implemented effectively, a “next generation” CHW platform could represent one of the most impactful investments available to governments and donors, not only in the fight against TB but also in efforts to improve population health more broadly (2).

By providing immunizations, nutrition education, pneumonia and diarrhea case management, and other maternal and child health support, CHWs play a critical role in promoting child survival. They can also prevent premature deaths among adults from control of non-communicable diseases. If these interventions achieve high levels of coverage, they have the potential to prevent millions of deaths every year (2, 28). Investments in CHWs can and should be considered part of national efforts to improve population health and spur economic growth.

## How CHWs can resolve the tension between vertical disease control programs and comprehensive, integrated primary health care

For almost a half century now, since the Declaration of Alma-Ata in 1978 (29), arising from the International Conference on Primary Health Care, and since the 1979 landmark paper by Walsh and Warren (30), there have been competing visions for how PHC can best be implemented. While the Declaration of Alma-Ata recognized the importance of vertical, disease-specific approaches, its focus was primarily on a comprehensive and participatory approach: defining PHC as “essential health care based on practically, scientifically sound and socially accessible methods and technology made universally accessible to individuals and families in the community through their full participation and at a cost that the community and country can afford to maintain at every stage of their development in the spirit of self-reliance and self-determination” (29). While Walsh and Warren viewed the broader concept of PHC as defined at Alma-Ata as a worthwhile and legitimate long-term approach, they did not consider it to be the best approach for the short-term. In fact, the full title of their article is “Selective Primary Health Care: An Interim Strategy for Disease Control in Developing Countries” (30). They stated upfront that, while comprehensive PHC is the best solution to improving the health of populations, “its very scope makes it unattainable because of the cost and numbers of trained personnel required” (30) (p. 967).

Walsh and Warren endorsed the concept of selective PHC in which programmatic efforts are focused primarily on epidemiological priorities through interventions that have been demonstrated to be cost-effective. By their nature, selective PHC approaches tend to be top-down, driven by centralized technical experts with less concern about broader community health needs. This approach tends to be composed of “interventions into the lives of other people,” as Randall Packard described it in his magisterial history of global public health (31).

The power of selective PHC is its capacity to focus intensively on priority problems and measure results in relatively short periods of time. The power of the Alma-Ata version of PHC is its engagement of the community, including training community members to become CHWs; in the provision of basic and essential services as close to the community as possible; and in responding to the felt needs of the community. Separate programs for family planning, immunizations, child survival, HIV/AIDs, malaria, as well as for TB are all examples of selective PHC interventions.

A “next generation” of CHW programs would make it possible to maintain the strength of all of these vertical disease programs through an integrated community platform of CHWs who are in regular contact with all households. Moreover, by providing CHWs with the skills to provide more comprehensive PHC, the broader health needs of the community can be addressed at the same time. Embedding TB and other vertical programs within a strong CBPHC platform will make it possible to do more with less – a necessity in the era of reduced donor aid which we are now entering (32, 33).

## Conclusion

A stronger commitment to CBPHC and CHWs is now needed, given the slow progress at present in reaching the health-related SDGs. Building on BRAC’s experience in Bangladesh in which a CHW visits every home monthly to provide a broad range of basic services while at the same providing TB-related services with excellent results, a “next generation” of CHWs has the potential to accelerate progress not only in the control of tuberculosis but in addressing other priority health issues as well. Because of the task-sharing that would occur, savings in health care costs would accrue as well, making this a highly cost-effective intervention in the long term. As Wally and others noted, “The emphasis has to shift from showing immediate results from single interventions to creating, integrated, long-term, sustainable health systems, which can be built from a more selective primary health-care start” (34). Routine home visits from well-trained and well-supported CHWs can make this possible.

## Data Availability

The original contributions presented in the study are included in the article. Further inquiries can be directed to the corresponding author.

## References

[1] BRAC BRAC: Who We Are. (2026). Available online at: http://www.brac.net/who-we-are (Accessed April 16, 2026).

[2] The Johns Hopkins Team, the UNICEF Team, and the Expert Panel for the External Expert Panel for the “Next Generation” of Community Health Workers in Low-Income and Lower Middle-Income Countries. The Investment Case for Community-based Primary Health Care and Community Health Workers in Low-Income and Lower Middle-Income Countries. New York, New York, United States: UNICEF (2026).

[3] United Nations. The Sustainable Development Goals Report 2024. (2024). Available online at: https://unstats.un.org/sdgs/report/2024/The-Sustainable-Development-Goals-Report-2024.pdf (Accessed April 16, 2026).

[4] WHO. Global Tuberculosis Report 2025. (2025). Available online at: https://www.who.int/teams/global-programme-on-tuberculosis-and-lung-health/tb-reports/global-tuberculosis-report-2025 (Accessed April 16, 2026).

[5] CDC. Tuberculosis. (2025). Available online at: https://www.cdc.gov/global-hiv-tb/media/pdfs/2025/03/2025_DGHT-TB-Overview-Factsheet.pdf?utm_source=copilot.com (Accessed April 16, 2026).

[6] Reid MJA Arinaminpathy N Bloom A Bloom BR Boehme C Chaisson R . Building a tuberculosis-free world: the lancet commission on tuberculosis. Lancet. (2019) 393:1331–84. doi: 10.1016/S0140-6736(19)30024-8, 30904263

[7] Sinha P Shenoi SV Friedland GH. Opportunities for community health workers to contribute to global efforts to end tuberculosis. Glob Public Health. (2020) 15:474–84. doi: 10.1080/17441692.2019.1663361, 31516079 PMC7028491

[8] WHO Global Gains in Tuberculosis Response Endangered by Funding Challenges (2025). Available online at: https://www.who.int/news/item/12-11-2025-global-gains-in-tuberculosis-response-endangered-by-funding-challenges (Accessed April 16, 2026).

[9] O'Donovan J Baskin C Stansert Katzen L Ballard M Kok M Jimenez A . Costs and cost-effectiveness of community health worker programs focussed on HIV, TB and malaria infectious diseases in low- and middle-income countries (2015-2024): a scoping literature review. PLoS Glob Public Health. (2025) 5:e0004596. doi: 10.1371/journal.pgph.0004596, 40343952 PMC12063845

[10] O'Donovan J Kumar MB Ballard M O’ Donovan J Mchenga M Martin L . Costs and cost-effectiveness of integrated horizontal community health worker programmes in low- and middle-income countries (2015-2024): a scoping literature review. BMJ Glob Health. (2025) 10:1–11. doi: 10.1136/bmjgh-2024-017852, 40701612 PMC12306334

[11] Knowles M Crowley AP Vasan A Kangovi S. Community health worker integration with and effectiveness in health care and public health in the United States. Annu Rev Public Health. (2023) 44:363–81. doi: 10.1146/annurev-publhealth-071521-031648, 37010928

[12] Perry HB Bang A Hodgins S Ballard M Sachs JD Muluneh AZ The Potential of a "Next Generation" of Community Health Workers to Accelerate Progress in Reaching Universal Health Coverage and Health for All. San Francisco, California, United States: PLOS Global Public Health (2026).

[13] Perry HB Chowdhury M Were M LeBan K Crigler L Lewin S . Community health workers at the dawn of a new era: 11. CHWs leading the way to "health for all". Health Res Policy Syst. (2021) 19:111. doi: 10.1186/s12961-021-00755-5, 34641891 PMC8506098

[14] Chowdhury AM Bhuiya A Chowdhury ME Rasheed S Hussain Z Chen LC. The Bangladesh paradox: exceptional health achievement despite economic poverty. Lancet. (2013) 382:1734–45. doi: 10.1016/S0140-6736(13)62148-0, 24268002

[15] Perry HB Chowdhury AMR. Bangladesh: 50 years of advances in health and challenges ahead. Global Health Sci Pract. (2024) 12:e2300419. doi: 10.9745/GHSP-D-23-00419, 38233096 PMC10906562

[16] Zaman K Hossain S Banu PC Quaiyum MA Barua PC Salim MA . Prevalence of smear-positive tuberculosis in persons aged >/= 15 years in Bangladesh: results from a national survey, 2007-2009. Epidemiol Infect. (2012) 140:1018–27. doi: 10.1017/S0950268811001609, 21880168

[17] Faiz M Rahman M Husain M Sharif A Wahra G Faisel A. "Disasters, epidemics and public health". In: Chowdhury A Ahmed Y Islam K Moral S, editors. 50 Years of Bangladesh Advances in Health. Dhaka, Bangladesh: University Press Limited in association with Bangladesh Health Watch (2023). p. 80–94.

[18] WHO Tuberculosis Profile: Bangladesh (2025). Available online at: https://worldhealthorg.shinyapps.io/tb_profiles/?_inputs_&tab=%22charts%22&lan=%22EN%22&iso3=%22BGD%22&entity_type=%22country%22 (Accessed April 16, 2026).

[19] Chowdhury AM Chowdhury S Islam MN Islam A Vaughan JP. Control of tuberculosis by community health workers in Bangladesh. Lancet. (1997) 350:169–72. doi: 10.1016/S0140-6736(96)11311-8, 9250184

[20] Joardar T Javadi D Gergen J Perry H. "The BRAC Shasthya Shebika and Shasthya Kormi community health Workers in Bangladesh". In: Perry H, editor. Health for the People: National Community Health Programs from Afghanistan to Zimbabwe. Washington. DC: USAID/Jhpiego/Maternal and Child Survival Program (2020)

[21] Joardar T Javadi D Gergen J Perry H. "The government family welfare assistants, health assistants, and community health care providers in Bangladesh". In: Perry H, editor. National Community Health Programs: Descriptions from Afghanistan to Zimbabwe. Washington, DC: USAID/Jhpiego/Maternal and Child Survival Program (2020)

[22] BRAC. Annual Report 2025: Tuberculosis Control Project, BRAC Health Programme. Dhaka, Bangladesh: BRAC (2025).

[23] BRAC. BRAC Annual Report 2023: National Tuberculosis Control Programme. Dhaka, Bangladesh: BRAC (2024).

[24] Islam MA Wakai S Ishikawa N Chowdhury AM Vaughan JP. Cost-effectiveness of community health workers in tuberculosis control in Bangladesh. Bull World Health Organ. (2002) 80:445–50. doi: 10.1590/S0042-9686200200600007 12132000 PMC2567545

[25] Chowdhury A Perry H. "NGO contributions to community health and primary health care: case studies on BRAC (Bangladesh) and the comprehensive rural health project, Jamkhed (India)". In: Oxford Research Encyclopedia, Global Public Health. New York, NY: Oxford University Press USA (2020)

[26] Uddin M George J Jahan S Shams Z Haque N Perry H. Learnings from a pilot study to strengthen health care services: the community-clinic-centered health service model in Barishal District, Bangladesh. Glob Health Sci Pract. (2021) 9:S179–89. doi: 10.9745/GHSP-D-20-00466 33727329 PMC7971368

[27] Lateef S. Bangladesh’s ambitious new health plans. Lancet. (2026) 407:933–4. doi: 10.1016/s0140-6736(26)00458-7

[28] Chou VB Friberg IK Christian M Walker N Perry HB. Expanding the population coverage of evidence-based interventions with community health workers to save the lives of mothers and children: an analysis of potential global impact using the lives saved tool (LiST). J Glob Health. (2017) 7:020401. doi: 10.7189/jogh.07.020401, 28959436 PMC5592116

[29] WHO, UNICEF Declaration of Alma-Ata (1978). Available online at: https://cdn.who.int/media/docs/default-source/documents/almaata-declaration-en.pdf?sfvrsn=7b3c2167_2 (Accessed April 16, 2026).

[30] Walsh JA Warren KS. Selective primary health care: an interim strategy for disease control in developing countries. N Engl J Med. (1979) 301:967–74. 114830 10.1056/NEJM197911013011804

[31] Packard R. A History of Global Health: Interventions into the Lives of Other People. Baltimore, MD: Johns Hopkins University Press (2016).

[32] WHO Tuberculosis and Primary Health Care: Synergies and Opportunities Toward Universal Health Coverage: Policy Brief (2025). Available online at: https://www.who.int/publications/i/item/9789240111295 (Accessed April 16, 2026).

[33] Pai M Heitkamp P Pillay Y. Integration of tuberculosis services within primary health care: converting challenges into opportunities. Lancet Prim Care. (2025) 1:1–3. doi: 10.1016/j.lanprc.2025.100056

[34] Walley J Lawn JE Tinker A de Francisco A Chopra M Rudan I . Primary health care: making Alma-Ata a reality. Lancet. (2008) 372:1001–7. doi: 10.1016/S0140-6736(08)61409-9, 18790322

